# Electrochemical Energy Storage Properties of High-Porosity Foamed Cement

**DOI:** 10.3390/ma15072459

**Published:** 2022-03-26

**Authors:** Changshun Zhou, Qidong Wang, Congyan Zhang

**Affiliations:** Yuanpei College, Shaoxing University, Shaoxing 312000, China; zcs_forever@163.com (C.Z.); congyanzhang@usx.edu.cn (C.Z.)

**Keywords:** high-porosity, foamed cement, electrochemical, energy storage

## Abstract

Foamed porous cement materials were fabricated with H_2_O_2_ as foaming agent. The effect of H_2_O_2_ dosage on the multifunctional performance is analyzed. The result shows that the obtained specimen with 0.6% H_2_O_2_ of the ordinary Portland cement mass (PC0.6) has appropriate porosity, leading to outstanding multifunctional property. The ionic conductivity is 29.07 mS cm^−1^ and the compressive strength is 19.6 MPa. Furthermore, the electrochemical energy storage performance is studied in novel ways. The PC0.6 also shows the highest areal capacitance of 178.28 mF cm^−2^ and remarkable cycle stability with 90.67% of initial capacitance after 2000 cycles at a current density of 0.1 mA cm^−2^. The superior electrochemical energy storage property may be attributed to the high porosity of foamed cement, which enlarges the contact area with the electrode and provides a rich ion transport channel. This report on cement–matrix materials is of great significance for large scale civil engineering application.

## 1. Introduction

In the energy consumption structure, building energy consumption has accounted for a large part of the total social energy consumption, and this proportion is expected to increase with the continuous improvement requirements of people for the quality of life. Therefore, building energy efficiency is considered to be a key area to achieve carbon emission reduction targets. While the establishment of building energy saving equipment and management systems need energy supply and storage equipment. Common structural energy storage systems include batteries and supercapacitors, which store electrical energy only, an external packaging is required to ensure mechanical integrity. By contrast, in a structural energy storage system, structural electrodes and structural electrolytes themselves are designed to achieve electrochemical performance and to withstand mechanical loads simultaneously, so that they reach a reduction in the weight and volume of the entire system [1,2,3]. Therefore, a structural energy storage system is a multi-functional energy storage system with mechanical load and electrochemical energy storage functions, which have aroused great interest for research in fields of aerospace, automotive, and architectures [4].

The concept of structural supercapacitors was first put forward by Luo and Chung [5], who reported a thin device made of unidirectional CF prepreg layers separated by a paper dielectric. O’Brien [6] defined and evaluated the multifunctional performance of the device, which was conductive to the multifunctional design of structural supercapacitors. Currently developed multifunctional structural supercapacitors (MSS) mainly consist of carbon fiber (CF) braided into two electrodes separated by a glass fiber (GF) separator, and all electrodes were stuck on a solid polymer electrolyte (SPE) matrix. The GF separator is ionically conductive but electronically non-conductive; in other words, ions can be transferred from one side to the other [7]. The SPE matrix owns both mechanical and electrical properties, which is critical to the supercapacitor. The SPE matrix is mainly composed of highly structural polymers and ionic liquids (ILs) or ionic salts (lithium salts, etc.), making it ionic conductive [8,9,10]. The high ionic conductivity of structural supercapacitors is beneficial to electron transfer and motion, thus improving the areal capacitance of structural supercapacitors.

Foamed cement-based materials with high porosity have been extensively applied to thermal insulation and shock absorption barriers in civil infrastructures due to their low thermal conductivity and good seismic performance [11,12]. At present, most of the research on porous cement-based materials mainly focuses on the influence of foaming agent, water cement ratio and other factors on thermal resistance and porosity. For example, Panesar et al. [13] studied three different foaming agents, one of which was a protein-based agent, and the other two are synthetic agents. The results show that the type of foaming agent has significant influence on the thermal insulation coefficient, microstructure of the bubbles, and thermal resistance of foam cement-based materials. Nambiar et al. [14] investigated the influence of water content on pore morphology. Falliano et al. [15] found that the foaming agent had a great influence on the compressive strength in the low-density range. However, the electrochemical energy storage performance of porous cement materials has been rarely researched.

In this paper, we prepared the high-porosity foamed cement using ordinary Portland cement as cementitious material and H_2_O_2_ as foaming agent. The effect of H_2_O_2_ mass content on electrochemical performance and multifunctionality of the foam cement were studied in detail.

## 2. Materials and Methods

### 2.1. Materials

Ordinary Portland cement with type of 42.5 R was bought from Hailuo Cement Company, City, China. Hydrogen peroxide (H_2_O_2_) and Potassium hydroxide (KOH) were obtained from Sinopharm Chemical Reagent Co., Ltd. (Shanghai, China) Foamed nickel with mass per unit area of 320 ± 20 g m^−2^ was purchased from Kunshan Kuangxun Electronics Co. (Kunshan, China). The GO suspension with a concentration of 22 mg mL^−1^ was self-made in our laboratory. Deionized water was used for specimen preparation.

### 2.2. Specimen Preparation

Cement pastes’ specimens with different mix design were prepared in this research. KOH was dissolved in a certain amount of deionized water, and then the solution was added to the cement and stirred evenly. Finally, H_2_O_2_ was added and stirred for 30 s rapidly. Then, the mixture was poured into three custom cube molds of 30 mm × 30 mm × 30 mm for compressive strength and another custom cube mold of 10 mm × 10 mm × 10 mm with two stainless steels inserted into both edges for the ionic conductivity test. The specimens were cured in a curing chamber until test. The properties of the specimens are shown in Table 1. The dosages of H_2_O_2_ were 0%, 0.2%, 0.4%, 0.6%, 0.8%, and 1.0% of the mass of the ordinary Portland cement, respectively. The water–cement ratio is kept at 0.4, and the KOH concentration is 2 M. The cement specimens are labeled as PC0, PC0.2, PC0.4, PC0.6, PC0.8, and PC1.0.

### 2.3. Assemble of Energy Storing Devices

The rGO/Ni foam electrode was firstly prepared. Briefly, GO suspension was coated on nickel foam and then dried at 60 °C for 2 h. The GO/Ni foam was put into a hydrothermal reaction vessel filled with 60 mL deionized water and maintained at 180 °C for 12 h. After cooling to room temperature, take out the rGO/Ni foam electrode, and clean it with ethanol and deionized water three times, respectively. The rGO/Ni foam electrode was dried at 60 °C for 6 h before being used. The weight of rGO on nickel foam was about 1.2 mg cm^−2^. Then, the above cement paste was placed between two slices of rGO/Ni foam electrodes with a size of 1 cm × 3 cm and a distance of 1 cm. The samples were kept in a curing room until testing.

### 2.4. Material Characterization

The micromorphology of the foamed cement samples was observed using a field emission scanning electron microscope (FE-SEM, ZEISS Gemini 300, Zeiss, Karl, Germany). A power X-ray diffraction pattern (XRD, Rigaku Ultima IV, Akishima, Tokyo, Japan) was conducted to analyze the chemical components. Mercury intrusion porosimetry (MIP, Mike 9620, Atlanta, GA, USA) was applied to examine the porosity of samples.

### 2.5. Electrochemical and Mechanical Measurements

The electrochemical properties of the synthesized cement pastes and assembled energy storing devices were studied using two-electrode cells on a CHI660E electrochemical workstation. Cyclic voltammetry (CV) and galvanostatic charge/discharge (GCD) tests were performed at a potential range of (−0.5) −0.5 V. Electrochemical impedance spectroscopy (EIS) analysis of the cement pastes was collected in the frequency range from 0.01 Hz and 10^5^ Hz. The intercept between the EIS curve and the *x*-axis at high frequency is regarded as the bulk resistance of the cement. The ionic conductivity of cement pastes was obtained from the following Equation (1):

(1) σ=d/(S×Rb)

where *d* represents the thickness of the cement between two electrodes (cm), *Rb* represents the bulk resistance of the cement slurry (Ω), and *S* refers to the contact area of rGO/Ni foam electrode and cement electrolyte (cm^2^).

The corresponding areal capacitances *C* (mF cm^−2^) of devices were obtained from GCD curves by Equation (2):

(2) C=IΔt/SΔV

where ∆*V* represents the working voltage window (*V*), I is the current (mA), *S* represents the contact area of rGO/Ni foam electrode with cement (cm^2^), and ∆*t* represents the discharge time (s).

The energy density and power density (*P*) of the devices can be calculated according to Equations (3) and (4), respectively [16]:

(3) $$E=\frac{C\Delta V^{2}}{2}$$

(4) $$P=\frac{E\times 3600}{\Delta t}$$

where *C* represents the specific capacitance of the device (F g^−1^), ∆*V* is the working voltage window (V) during the discharge process, and ∆*t* is the time of discharge (s).

The mechanical strength of cement slurry was conducted on the JES-300 concrete compressive strength tester at a loading rate of 2.4 kN s^−1^.

## 3. Results

**Figure 1:**
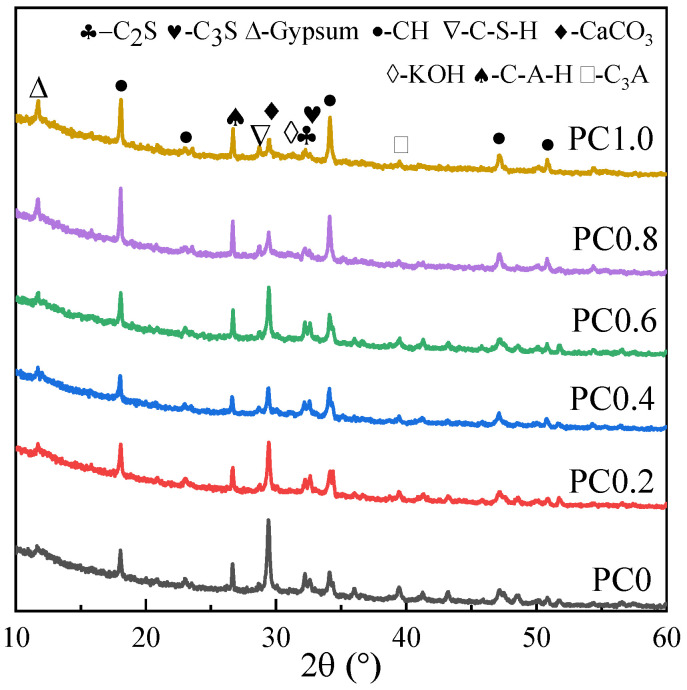
XRD patterns of various cement specimens.

Figure 1 displays the XRD patterns of various cement specimens. The hydration products in cement specimens are mainly Ca (OH)^2^, gypsum, C_3_S, C_2_S, hydrated calcium silicate (C-S-H), and calcium carbonate caused by carbonization, proving the composition of cement [17,18]. It is noted that, with the addition of H_2_O_2_, the characteristic peak value corresponding to CH in the samples is significantly enhanced, which may be caused by the inhibition of H_2_O_2_ on cement hydration.

**Figure 2:**
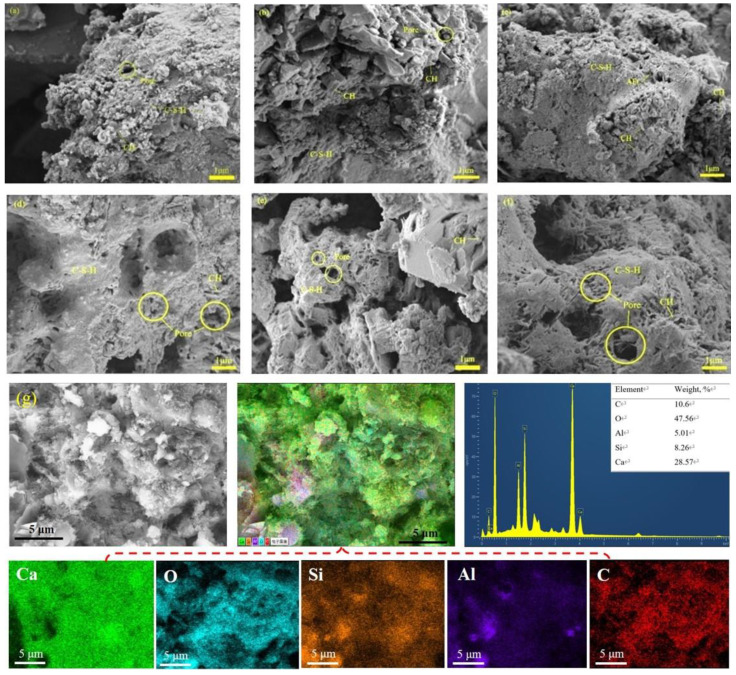
SEM images of various cement specimens, (**a**) PC0; (**b**) PC0.2; (**c**) PC0.4; (**d**) PC0.6; (**e**) PC0.8; (**f**) PC1.0; and (**g**) EDS elemental mapping of PC0.6.

In general, the microstructure of cement slurry has a great influence on its properties. It can be seen from Figure 2 that the microstructure of specimens is mainly composed of hydration gels, hydration products (CH), pores, and a few microcracks. The microstructures of the control group and the specimens containing H_2_O_2_ are obviously different. A few CH crystals and disconnected pores exist in the PC0 sample, but a lot of C-S-H gels are connected and covered with each other to form a relatively dense microstructure. In contrast, with the addition of H_2_O_2_ (Figure 2b–d), part of C-S-H gels in the samples are distributed in blocks, and the pore number increased, making the microstructure looser. When the H_2_O_2_ content further increases (Figure 2e,f), the number of pores in the samples increases significantly, especially that of large pores, and the pores are interconnected. The porous structure helps to shorten the pathway for transporting the ions, thus improving the electrochemical performance. The EDX analysis in Figure 2g also displays the presence of calcium, oxygen, silicon, aluminum, and carbon, homogenously distributed on the surface of the PC0.6 electrolyte.

**Figure 3:**
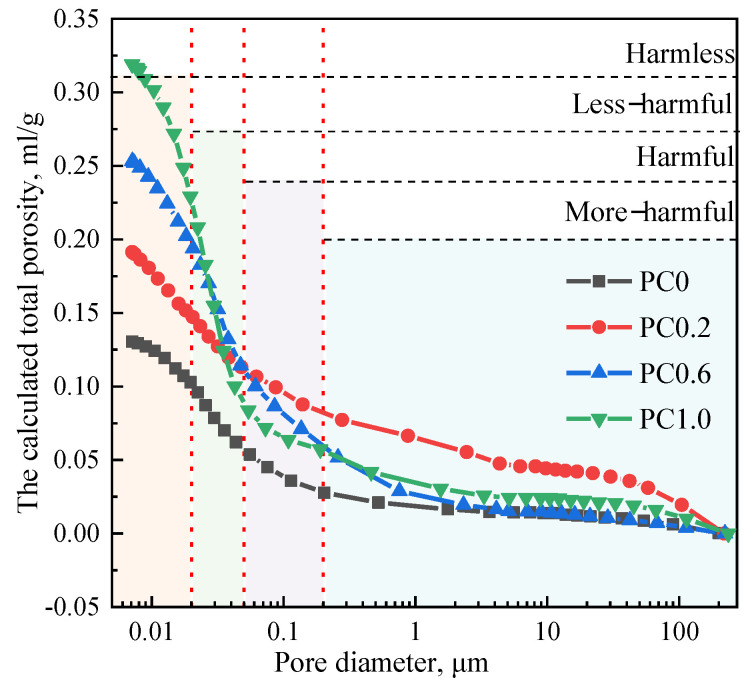
Pore size distribution curves of various cement specimens.

Pore structure, such as the porosity and pore size distribution, plays a decisive role in properties of cement slurry. Figure 3 shows the cumulative porosities of cement specimens tested by the MIP, and the corresponding detailed pore size distribution is shown in Table 2. As seen from the figure, the total porosity of samples increases with the increase of H_2_O_2_ content. It is well known that the pore can be divided into four categories according to its influence on cement materials: harmless pore with the diameter less than 20 nm, less harmful pore with the diameter of 20–100 nm, harmful pore with the diameter of 100–200 nm, and a multi-harmful pore with the diameter greater than 200 nm [19]. Previous studies noted that, according to the different effect of pore size on concrete properties, the pores with diameters more than 100 nm are defined as harmful pores [20,21].

**Figure 4:**
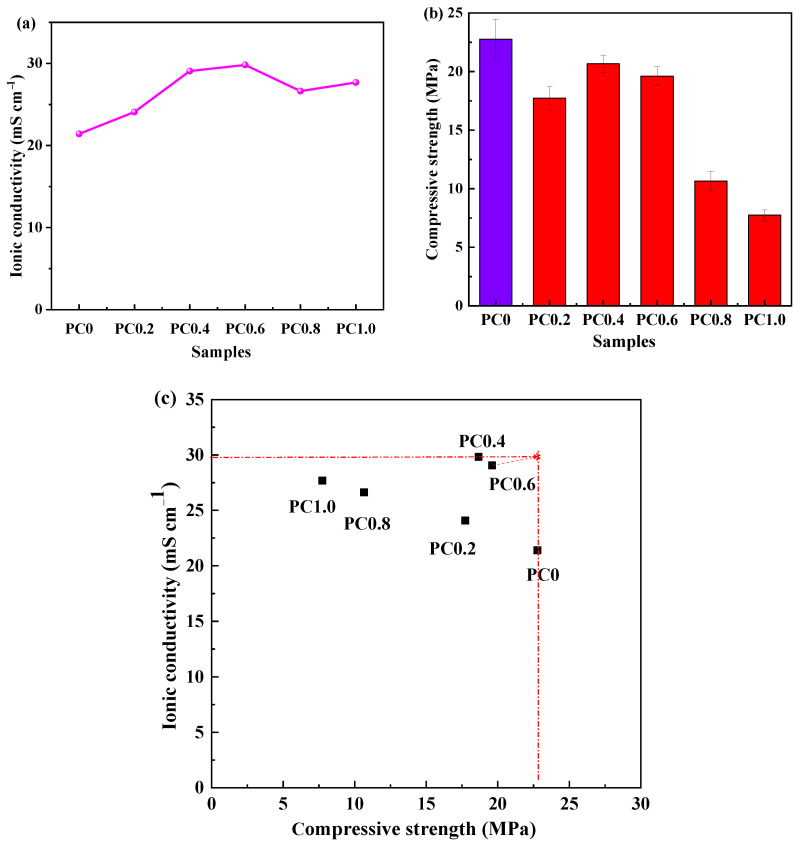
(**a**) Ionic conductivity; (**b**) compressive strength; and (**c**) multifunctional performance of different foamed porous cement specimens.

In order to characterize the ionic conduction of the foamed porous cement slurry, Figure 4a shows the ionic conductivity of various specimens. The ionic conductivity of pure cement slurry is 21.41 mS cm^−1^. After adding the foaming agent H_2_O_2_, the ionic conductivity increases from 12.52% to 39.28% due to the increase of pore number. Moreover, the PC0.6 shows the highest ionic conductivity, which is 29.82 mS cm^−1^. The ionic conductivity is a key parameter to determine the power and energy density of supercapacitors. The high ionic conductivity is attributed to the porous network structure within the foamed cement, which accelerates ion conduction [22]. However, by further increasing the amount of H_2_O_2_, the ionic conductivity was decreased due to the volume collapse of cement slurry and blocking the ions’ way. Figure 4b depicts the compressive strength of cement slurry. The compressive strength of pure cement is 22.8 MPa. After adding the H_2_O_2_, the compressive strength of the sample declines, but not much before the H_2_O_2_ content exceeds 0.4%. Its value is in the range from 17.7 to 18.7 MPa, while the compressive strength of foam porous cement shows a decreased trend when the H_2_O_2_ content is between 0.4–1.0%. The main reason for this phenomenon may be that, when the H_2_O_2_ content is lower than 0.4%, the amount of foaming is small, and the volume expansion of the materials is relatively small, thus containing more materials in the same volume, and the compressive strength is better. However, when the H_2_O_2_ content is about 0.4%, the compressive strength increased to the maximum 19.6 MPa. As the H_2_O_2_ content increases from 0.4% to 1.0%, the expansion volume of cement slurry increases in the foaming process, and the foam becomes unstable and eventually bursts. Therefore, the compressive strength decreases [23].

Figure 4c shows the multifunctionality analysis of various foamed porous cement specimens, demonstrating ionic conductivity as a function of compressive strength. It can be found that the optimal point is required with the highest ionic conductivity and the highest compressive strength in all samples. The closer the distance to the optimal point, the better the multifunctional performance of the foamed cement. The analysis result shows that the optimum foamed porous cement for energy storge is PC0.6 with an ionic conductivity of 29.07 mS cm^−1^ and a compressive strength of 19.6 MPa, which is comparable to those of other solid devices, as shown in Table 3.

**Figure 5:**
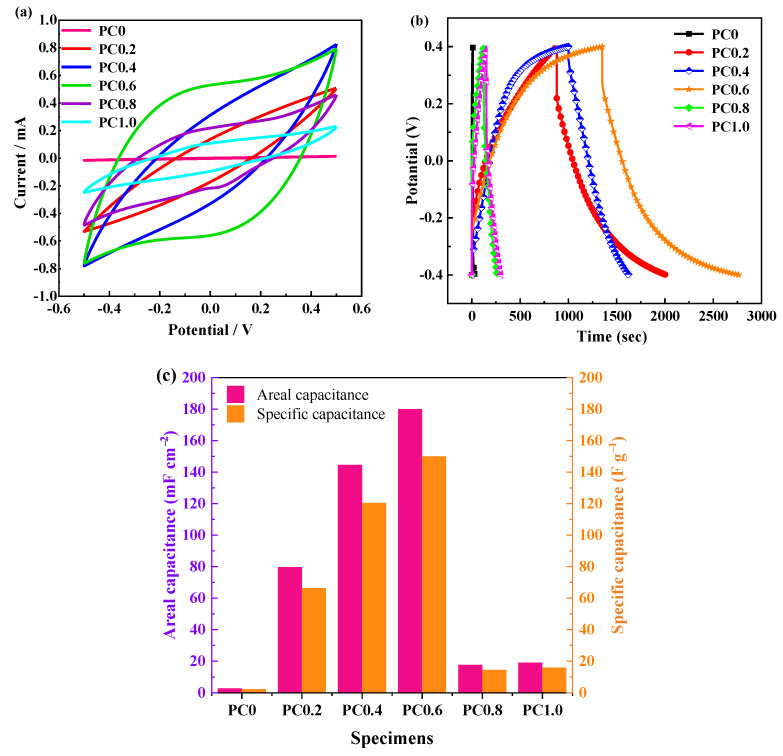
(**a**) CV curves; (**b**) GCD curves; and (**c**) the corresponding areal capacitances and specific capacitance of devices based on different cement pastes.

To further study the potential electrochemical properties of the foamed porous cement slurry, solid devices were assembled with rGO/Ni foam electrodes. The CV curves at a scan rate of 1 mV/s are plotted in Figure 5a. The CV curves of all specimens are nearly a rectangular shape, suggesting electric double-layer capacitance behavior. The CV curve area of each specimen is different at the same scanning rate. The area of PC0.6 is the largest, implying the highest capacitance of PC0.6 because the area of CV is proportional to the capacitance [30].

Figure 5b exhibits the GCD curves of different foamed porous cement. The quasi-triangle shapes demonstrate electric double-layer capacitance characteristics. The corresponding areal capacitation and specific capacitance are presented in Figure 5c. It can be seen that the PC0.6 solid electrolyte exhibits the highest areal capacitation of 179.98 mF cm^−2^ and specific capacitance of 150.0 F g^−1^. Therefore, PC0.6 has the optimal electrochemical energy storage performance. This may be attributed to the abundant porosity, which is conducive to ion transport and conduction.

**Figure 6:**
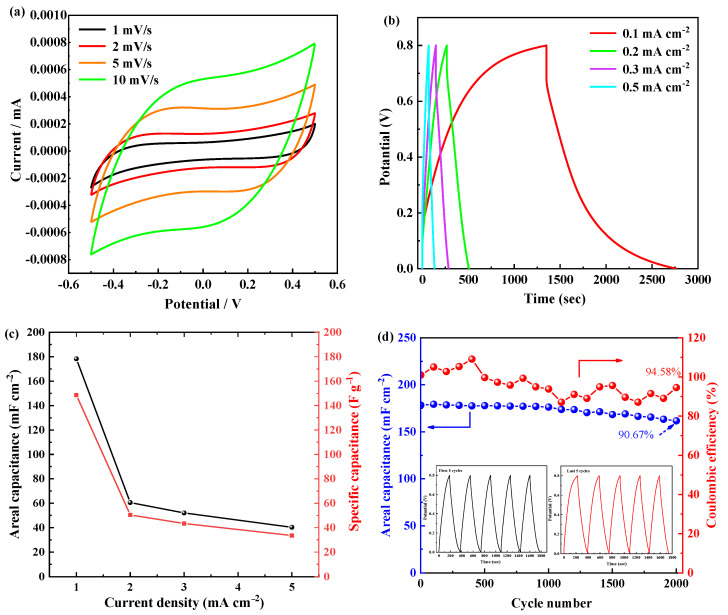

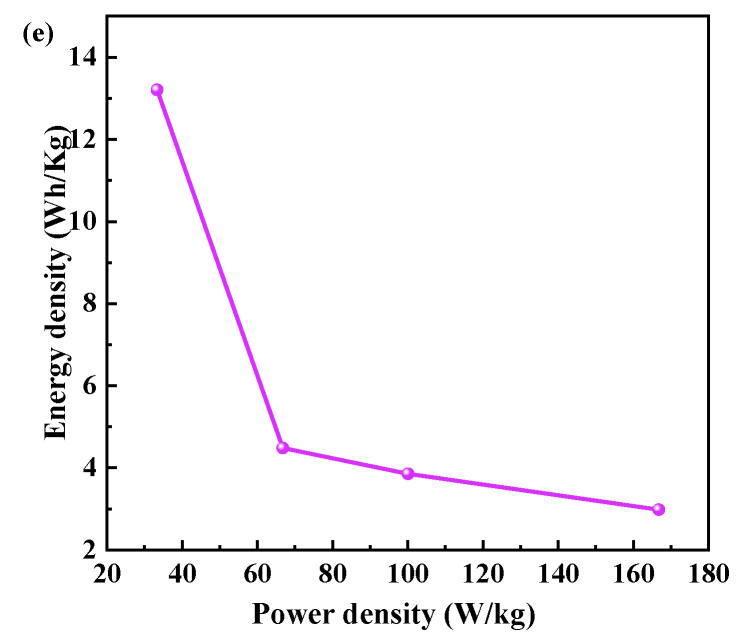
(**a**) CV curves at different scan rates; (**b**) GCD curves at various current density; (**c**) variation of areal capacitance vs. current densities; (**d**) cycling stability and coulombic efficiency for 2 × 10^3^ GCD cycles, and (**e**) Ragone plot of the device based on PC0.6.

Electrochemical performance of the optimal PC0.6 material was illustrated in Figure 6. The CV curves at various scan rates of PC0.6 are displayed in Figure 6a, which reveals the EDLC behavior. The CV curve area increases with the increase of scan rate, indicating that the resultant areal capacitance increases. Due to the increase in scanning rate, the current increases and electron transfer is better [31].

The galvanostatic charge/discharge curves of PC0.6 tested at different low current densities of 0.1, 0.2, 0.3, and 0.5 mA cm^−2^ are shown in Figure 6b. As the current density increases from 0.1 to 0.5 mA cm^−2^, the discharge time decreases gradually. The device with PC0.6 exhibits quasi-triangle curves at different current densities demonstrating EDLC characteristics. The calculated areal and specific capacitances from the charge/discharge curves are shown in Figure 6c. The areal capacitances at 0.1, 0.2, 0.3, 0.5 mA cm^−2^ are 178.28, 60.52, 52.06, and 40.28 mF cm^−2^, respectively. The specific capacitance of the device with PC0.6 varies from 148.57 F g^−1^ to 33.56 F g^−1^ as the charge/discharge current density increases from 0.1 to 0.5 mA cm^−2^. Figure 6d shows the cyclic stability test of the solid device based on PC0.6. It can be clearly observed that the areal capacitance and the coulombic efficiency of the device with PC0.6 remains 90.67% of initial capacitance and 94.58% after 2000 cycles at 0.1 mA cm^−2^, illustrating outstanding cycle stability of the solid device. The first five GCD cycles reveal a negligible difference when compared with the last five curves as displayed in the inset of Figure 6d. The superior cycling performance may be ascribed to the porous structure of cement slurry, providing a large number of transmission paths for ions. More importantly, the PC0.6 device can deliver a high energy density of 13.21 kW kg^−1^ with the corresponding power density of 33.36 Wh kg^−1^. The power density is up to 166.76 Wh kg^−1^ when the energy density is 2.98 kW kg^−1^. They are two crucial parameters in supercapacitor study [32].

## 4. Conclusions

Foamed porous cement materials with high porosity were fabricated by changing the foaming agent H_2_O_2_. Our work focused on researching the effect of the addition of H_2_O_2_ on the morphology, poor structure, and electrochemical energy storage performance of the foamed cement material. It displays that the optimum foamed porous cement for energy storge is PC0.6 with an ionic conductivity of 29.07 mS cm^−1^ and a compressive strength of 19.6 MPa. Simultaneously, the solid devices based on various foamed cement were assembled, and the electrochemical performance was measured. Among them, the device with PC0.6 exhibits high areal capacitance of 178.28 mF cm^−2^ and remarkable cycle stability with 90.67% of initial capacitance after 2000 cycles at 0.1 mA cm^−2^. The excellent electrochemical performance may be due to the high-porosity foamed cement that has more contact area with the electrode, and the porous structure provides a large number of ion transport paths.

## Figures and Tables

**Table 1:** Mix design of cement pastes.

Specimens	Cement/g	KOH/g	Water/g	H_2_O_2_/g
PC0	200	8.96	80	0
PC0.2	200	8.96	80	0.4
PC0.4	200	8.96	80	0.8
PC0.6	200	8.96	80	1.2
PC0.8	200	8.96	80	1.6
PC1.0	200	8.96	80	2.0

**Table 2:** The porosity in different pore size intervals of the cement specimens (mL/g).

Group	Total Porosity	Harmless	Less-Harmful	Harmful		More-Harmful
		0–20 nm	20–50 nm	50–100 nm	100–200 nm	>200 nm
PC0	0.1304	0.0283	0.0448	0.0189	0.0105	0.0278
PC0.2	0.1914	0.0437	0.0359	0.0164	0.0143	0.081
PC0.6	0.2532	0.057	0.085	0.0303	0.021	0.0591
PC1.0	0.3191	0.0926	0.1384	0.0232	0.0243	0.0404

**Table 3:** The ionic conductivity comparation of our device assembles by PC0.6 electrolyte with other solid devices.

Electrode	Electrolytes	Ionic Conductivity (mS.cm^−^¹)	Mechanical Property	Ref.
Polypyrrole	PVA-H₃PO₄	3.44	2 MPa (Tensile strength)	[24]
Activated carbon	PVA-H₃PO₄-Cellulose	0.104	-	[25]
Activated carbon	PVA-H₂SO₄	11.4	-	[26]
Graphene	Cement/KOH	1	9.85 MPa (Compressive strength)	[27]
SPE-CF	SPE	2	1.45 MPa (Compressive modulus)	[28]
CF fabric	PEGDGE/IL	28	9.78 MPa (Shear strength)	[9]
Graphene	Geopolymer-KOH	-	45 MPa (Compressive strength)	[29]
rGO/Ni foam	Foamed cement	29.07	19.6 MPa (Compressive strength)	This work

## Data Availability

The study did not report any data.
